# Low Back Pain Presentation and Management at the Emergency Department: Differences Between Older Adults Residing in the Community and Aged Care Homes

**DOI:** 10.1111/jep.70088

**Published:** 2025-04-11

**Authors:** Rod Ellem, Quinn Burling, Michel W. Coppieters, James Todd, Rowan Pickering

**Affiliations:** ^1^ Gold Coast Hospital and Health Service, Queensland Health, Gold Coast Southport Queensland Australia; ^2^ School of Health Sciences and Social Work, Griffith University Brisbane and Gold Coast Queensland Australia; ^3^ Queensland Health, Sunshine Coast Hospital and Health Service Birtinya Queensland Australia; ^4^ Amsterdam Movement Sciences, Faculty of Behavioural and Movement Sciences, Vrije Universiteit Amsterdam Amsterdam the Netherlands; ^5^ Bond Business School, Bond University Robina Queensland Australia; ^6^ Physiotherapy Department Faculty of Health Science, Bond University Robina Queensland Australia

**Keywords:** aged, back pain, emergency medical services, health services for the aged, homes for the aged

## Abstract

**Rationale:**

In healthcare systems without gatekeeper access to Emergency Departments (ED), the number of people presenting for low back pain (LBP) is increasing substantially. Low back pain presentations at the Emergency Department are rarely caused by serious underlying pathology, and management often deviates from practice guidelines. Older adults (≥ 65 years) constitute approximately 30% of all ED LBP presentations. Little is known about differences in presentation characteristics and ED management between older adults from aged care homes and those living in the community.

**Aims and Objectives:**

This study aimed to identify differences in presentation and management between people presenting at the ED for LBP from aged care homes versus those residing in the community.

**Methods:**

Retrospective observational study of routinely collected healthcare data and chart audits of older adults from aged care homes (*N* = 64) and age and sex‐matched community‐dwelling older adults (*N* = 64) presenting to ED for LBP.

**Results:**

Patients from aged care homes presented with more comorbidities (4 vs. 2), analgesic medication (84% vs. 70%) and polypharmacy (86% vs. 41%) and were more commonly admitted for ongoing analgesia or further diagnostic tests. Community‐dwelling older adults were more frequently admitted for Allied Health input. ED administration of opiates was high for both groups (81% aged care; 91% community‐dwelling). High rates of lumbar spine medical imaging (61% aged care; 50% community‐dwelling) resulted in few acute radiographic findings.

**Conclusions:**

Older patients presenting to ED for LBP receive similar management regardless of their residential status. Hospital management of both groups does not align with current published recommendations namely in respect to imaging and pain medication.

## 1 Introduction

Emergency Department (ED) presentations for low back pain (LBP) are prevalent in developed countries which have no gatekeeper access to ED. Despite significant research efforts and the implementation of guidelines regarding the management of LBP at the ED, the evidence‐based management of people with LBP is rarely achieved [1]. Variability in presenting patient characteristics and management of LBP is one reason contributing to the difficulty in applying evidence‐based guidelines to a more generalised population [2].

Up to 30% of all LBP presentations at the ED comprise of those over the age of 65 [3], with the prevalence of ED presentations increasing over the lifespan [4]. Older people with LBP have an increased length of hospital stay, higher likelihood of inpatient hospital admission from the ED and have higher rates of imaging and opiate use than the younger people with LBP [5]. With an ageing population across most developed countries [6], the prevalence of ED presentations in older persons are projected to rise further [4]. Several studies have highlighted that LBP in older adults contributes to greater rates of disability or loss of function than in younger adults [7, 8]. In some cases, this diminishes the ability of older adults' ability to perform activities of daily living or resulting in loss of independence [9].

For those living in an aged care home in Australia, the rates of ED presentations are further increased when compared to community‐dwelling older adults (938 presentations per 1000 vs. 449 per 1000 respectively, per year) [10]. The comorbid profile of these two groups typically differ, with aged care residents, at the time of admission to the aged care facility, having a higher prevalence of chronic disease burden affecting cognition, pain and disability, than community‐dwelling older adults [11]. This increased comorbid profile often acts as a catalyst for the change in care needs, necessitating admission to the aged care facility [12]. Other proposed reasons for the differences in presentation rates include the quality of care given in the aged care facility and the types of health services able to be delivered in the facility [10]. As such, the required acute care and ED management of aged care residents may differ to community‐dwelling older adults. Describing and profiling these differences is of value for the design of appropriate health service policy and to better inform clinicians delivering care to this population.

The aims of this study were to compare the patient characteristics and LBP characteristics, ED management, including medical imaging and medication use, and the prevalence and rationale for inpatient hospital admission for community‐dwelling older adults and aged care residents presenting to the ED for LBP. In agreement with the broader adult population presenting to the ED for LBP [10], we hypothesised that only a small proportion of older persons presenting for LBP would require emergency care for their condition. Despite anticipated differences regarding the level of independence and baseline comorbidities, we hypothesised that ED management of LBP would be largely similar in both populations.

## 2 Patients and Methods

### 2.1 Study Design

A retrospective observational study of routinely collected electronic medical record data and a retrospective electronic medical record chart audit were undertaken. Two age and sex‐matched cohorts consisting of community‐dwelling older adults and aged care residents presenting to the ED for LBP were analysed to determine differences in presentation, diagnosis and management of their LBP condition. Ethics approval was obtained from the Gold Coast Hospital and Health Service (GCHHS) Human Research Ethics Committee (reference no. LNR/2021/QGC/72060). As this was an anonymous and retrospective chart analysis, individual consent was not required.

### 2.2 Setting

The Gold Coast Hospital and Health Service is a public, multi‐site health service located on the Gold Coast, Australia, that services a region of approximately 635,000 residents [13]. The two participating EDs are located on the northern (Gold Coast University Hospital) and southern (Robina Hospital) ends of the Gold Coast.

### 2.3 Study Groups

Older adults (≥ 65 years of age) residing at aged care homes who presented at the ED between the 1 April 2019 and the 30 June 2021 because of LBP were considered for the aged care sample. For the sample of community‐dwelling older adults, we identified an age and sex‐matched patient from all people who attended the same ED departments for LBP during the same period.

### 2.4 Population Selection and Data Extraction

All data regarding the ED presentations of eligible participants were extracted by the GCHHS Health Analytics Department from the electronic medical records (FirstNet, Cerner, 2021). In addition, a chart audit of the individual patient's electronic medical record was undertaken by a trained data collector for non‐routine and qualitative data.

The selection criteria and processes were based on recent publications studying the same populations [5, 14]. Eligible patients were identified via a text search of all ED presentations from April 2019 until June 2021 for patients aged 65 years or older with a presenting complaint of ‘Back Pain’ via the GCHHS Data SQL server. These were further refined via diagnostic codes assigned to the patient whilst in the ED, which are standardised under the Systematised Nomenclature of Medicine Clinical Terms (SNOMED‐CT). Eligible diagnoses of those included in the study are listed in Appendix S1. The age cut‐off of 65 years and older was chosen to be consistent with previous research in this field and with the age classification of ‘older adult’ [15].

Aged care residents were identified by the patient's residential address matched to that of the residential addresses of aged care homes in the ED catchment areas. All older adults without a residential address of an aged care facility were categorised as community‐dwelling older adults. All eligible aged care residents were included in the study population. An age and sex‐matched sample of community‐dwelling adults presenting to the ED with LBP was obtained by a researcher, blinded to all non‐demographic data.

Routinely collected electronic medical record data and associated data captured from a chart audit of eligible patients were categorised as presentation variables, diagnosis and management variables. Presentation variables included: duration of symptoms, prescribed medications at the time of presentation, comorbid status and social factors, such as requirement of social support to maintain independence for community‐dwelling adults and whether the patient lived alone or with family. For these presenting variables, LBP was considered chronic if symptoms had been present for ≥ 12 weeks at the time of presentation [16]. Polypharmacy was defined as those patients presenting to ED with concomitant use of five of more scheduled medications daily [17]. Chronic comorbidities were defined as any chronic condition listed on the Cumulative Illness Rating Scale–Geriatric (CIRS‐G) [18]. At the time of ED presentation, patients were classified according to treatment acuity (i.e., the maximal waiting time for medical assessment and treatment) (Australasian Triage Scale, ATS category 1–5; Australian College of Emergency Medicine).

Management variables included any form of lumbar spine medical imaging undertaken during the ED presentation. The outcome of the imaging was categorised as acute imaging findings (e.g., a recent fracture or a previously not identified sinister cause of the patient's LBP); longstanding imaging findings (e.g., longstanding (degenerative) changes in the lumbar spine) or no positive imaging findings. Any additional diagnostic test, including blood pathology, urine analysis, electrocardiogram, chest X‐Ray and abdominal ultrasound, were included in the audit. Post‐discharge management variables included any analgesic medication prescription. Finally, in participants who were admitted to hospital, the clinician's reason for admission was recorded verbatim into the standardised data collection form and later analysed to identify three pre‐defined themes: ‘ongoing need for analgesia’, ‘further diagnostic tests’ and ‘Allied Health input’.

### 2.5 Statistical Analyses

IBM SPSS Statistics Version 27 [19] and R Studio [20] were used to perform all statistical analyses. *χ* ^2^ tests and Fisher's exact tests were used to test for statistically significant differences between the categorical presentation, diagnosis and management variables of aged care residents and community‐dwelling older adults where appropriate. The Mann–Whitney *U* test was used to test for statistically significant differences between the non‐categorical presentation and management variables of aged care residents and community‐dwelling older adults using a *p* < 0.05 to determine statistical significance. The initial analysis was performed by author Q. B., and verified by J. T., who has advanced expertise in healthcare analytics and statistical models.

## 3 Results

### 3.1 Sample Characteristics and Trends

From 1 April 2019 to 30 June 2021, there were 4433 presentations to one of the two GCHHS EDs for a primary complaint of LBP by persons aged 18 years and older. Of these, 1078 presentations were older adults, aged 65 years or older (24%). Of these older adults, there were 64 patients who resided at an aged care facility. Sixty‐four age and sex‐matched (±1 year) community‐dwelling older adults were identified from the same period for the purposes of this comparison.

### 3.2 Presentation Characteristics

Patient presenting variables for aged care residents and community dwelling older adults at the time of ED visit are presented in Table 1. Both groups had a median (IQR) age of 85 [10] years, with females accounting for 67% (*n* = 43) of all presentations. Mode of arrival showed high prevalence of ambulance use by both aged care residents (*N* = 60; 94%) and community‐dwelling older adults (*N* = 55; 86%). Most patients in either group (*N* = 62; 97%) were triaged as category ATS 3 or 4. Median (IQR) ED length of stay for aged care residents was 8 (8) hours and for community‐dwelling older adults it was 7 (10) hours (Table 1).

**Table 1:** Demographic and ED presentation characteristics.

	Aged care	Community‐dwelling	Test	Statistic	*p* value
Sex, female, *n* (%)	43 (67%)	43 (67%)	*χ* ^2^	0	1
Age (years), median (IQR)	85 (9)	85 (9)	Mann–Whitney *U*	0	1
Age range (years)	66–101	66–102			
Mode of arrival, *n* (%)			Fisher's exact		0.2412
Ambulance	60 (94%)	55 (86%)			
Walked in	4 (6%)	9 (14%)			
LBP diagnosis, *n* (%)			Fisher's exact		0.2676
Non‐specific LBP, *n* (%)	61 (95%)	57 (89 %)			
Radicular LBP, *n* (%)	1 (2%)	5 (8%)			
Serious LBP, *n* (%)	2 (3%)	2 (3%)			
Chronic LBP presentation, *n* (%)	34 (53%)	27 (42%)	*χ* ^2^	1.1	0.2883
Number of comorbidities, median (IQR)	4 (2)	2 (2)	Mann–Whitney *U*	3160	< 0.001
Baseline polypharmacy, *n* (%)	55 (86%)	26 (41%)	*χ* ^2^	31.6	< 0.001
Baseline analgesics, *n* (%)	54 (84%)	45 (70%)	*χ* ^2^	6.1	0.0137
Fall in recent history, *n* (%)	17 (27%)	6 (9%)	*χ* ^2^	5.3	0.0213
Lives alone, *n* (%)		13 (20%)			
Uses formal support services, *n* (%)		22 (34%)			
Initial ED pain score/10, median (IQR)	8 (2)	7 (5)	Mann–Whitney *U*	526	0.4745
Triage category, *n* (%)			Fisher's exact		0.3772
1	0 (0%)	0 (0%)			
2	2 (3%)	1 (1%)			
3	39 (61%)	31 (48%)			
4	23 (36%)	31 (48%)			
5	0 (0%)	1 (2%)			
Weekend presentations, *n* (%)	16 (25%)	13 (20%)	*χ* ^2^	0.2	0.6728
Presentation outside working hours, *n* (%)	25 (39%)	30 (47%)	*χ* ^2^	0.5	0.4751
ED length of stay (hours), median (IQR)	8 (8)	7 (10)	Mann–Whitney *U*	2110	0.7695
Admitted to hospital, *n* (%)	22 (33%)	20 (31%)	*χ* ^2^	0	1
Hospital length of stay (days), median (IQR)	3 (3)	3 (5)	Mann–Whitney *U*	200	0.9678
Consult to physiotherapy, *n* (%)	19 (30%)	28 (44%)	*χ* ^2^	2.2	0.1424
Additional diagnostic tests ordered, *n* (%)	42 (66%)	33 (51%)	*χ* ^2^	2.1	0.1511

The key differences in presentation variables identified between the groups were as follows: Aged care residents presented with more comorbidities (median (IQR) of 4 (2) vs. 2 (2); *p* < 0.001); higher rates of baseline polypharmacy (*N* = 55 (86%) vs. *N* = 26 (41%); *p* < 0.001) and baseline analgesic medications (*N* = 54 (86%) vs. *N* = 45 (70%); *p* = 0.0137). Residents from aged care facilities also had a higher prevalence of a recent falls history (*N* = 17 (27%) vs. *N* = 6 (9%); *p* = 0.0213) (Table 1).

### 3.3 ED Diagnosis

There were no statistically significant differences between aged care residents and community‐dwelling older adults for ED LBP diagnoses (*p* = 0.21). The most common diagnostic category was non‐specific LBP (aged care residents: *N* = 62 (97%), community‐dwelling older adults: *N* = 58 (91%)). There were very few diagnoses of radicular LBP (aged care residents: *N* = 1 (2%); community‐dwelling older adults: *N* = 5 (8%)).

### 3.4 Management Characteristics

Rates of admission to hospital from the ED were similar between groups. Aged care residents had admission rates of 33% (*N* = 21) in comparison to 31% (*N* = 20) for community‐dwelling older adults. Community‐dwelling older adults received more Physiotherapy consultation requests whilst in ED (44%) than aged care residents (30%). Median hospital length of stay was 3 days for both groups of patients for those admitted to a ward. The most common documented reason for hospital admission was for ongoing analgesia in both groups (aged care: 76%; community‐dwelling: 65%). The secondary reasons for admission included requests for further diagnostic testing (aged care: 38%; community‐dwelling: 20%) and Allied Health input, the latter notably requested more frequently for community‐dwelling older adults (80%), when compared to aged care residents (33%).

A non‐significant difference was observed in prevalence of ED lumbar spine imaging between community dwelling older adults (*N* = 32 (50%)) and residential aged care groups (*N* = 39 (61%)) (Table 2). Plain radiographs were the most frequently requested imaging modality (aged care: 47% vs. community‐dwelling: 44%). CT scans were the next most common (aged care: 33% vs. community‐dwelling: 14%), followed by MRI (aged care: 3% vs. community‐dwelling: 5%). Outcomes of imaging showed few acute findings for both groups (aged care: 15% vs. community‐dwelling: 13%). A high prevalence of chronic or degenerative changes on imaging were noted for both groups (aged care: 77% vs. community‐dwelling: 78%).

**Table 2:** Findings of individuals who received imaging.

	Aged care	Community‐dwelling	*χ* ^2^	*p* value
Received any form of imaging, *n* (%)	39 (61%)	32 (50%)	1.55	0.21
Acute positive imaging finding, *n* (%)	6 (15%)	5 (16%)	0.10	0.75
Chronic imaging finding, *n* (%)	30 (77%)	25 (78%)	0.80	0.37
No positive imaging finding, *n* (%)	3 (8%)	4 (13%)	0.15	0.70

A greater number of community‐dwelling adults overall received at least one analgesic medication (community‐dwelling: 98% vs. aged care: 86%). Opioid medications were the most common pain relief medication administered to both groups (aged care: 81% vs. community‐dwelling: 91%), followed by simple analgesics (aged care: 77% vs. community‐dwelling: 84%) (Table 3). Community‐dwelling older adults received a greater number of NSAIDs (39%) versus aged care residents (19%). The prevalence of patients who were prescribed new analgesic medications at discharge was similar between groups (aged care: 45% vs. community‐dwelling: 55%).

**Table 3:** Medication management of aged care residents and community‐dwelling older adults who presented to ED with LBP.

Medication received	Aged care	Community‐dwelling	*χ* ^2^	*p* value
Opiate, *n* (%)	52 (81%)	58 (91%)	2.33	0.13
Anti‐epileptic, *n* (%)	8 (13%)	14 (22%)	1.98	0.16
Oral corticosteroid, *n* (%)	6 (9%)	13 (20%)	3.04	0.08
Benzodiazepine analgesic, *n* (%)	5 (8%)	13 (20%)	4.12	0.04
NSAID, *n* (%)	12 (19%)	25 (39%)	6.43	0.01
Simple analgesic, *n* (%)	49 (77%)	54 (84%)	1.24	0.27
Any pain‐relieving medication, *n* (%)	55 (86%)	63 (98%)	6.94	0.008
New medication prescription at discharge, *n* (%)	29 (45%)	35 (55%)	1.33	0.25

Abbreviations: ED, emergency department; LBP, low back pain; NSAID, non‐steroidal anti‐inflammatory drug.

## 4 Discussion

This study aimed to identify differences in presentation and management between people presenting at the ED for LBP from aged care homes versus those residing in the community. Differences were revealed in both presenting characteristics, such as comorbid conditions at time of ED visit, and in management variables such as hospital admission rates and analgesic prescription in the ED. In addition, some management characteristics of the elderly patient within this study were different to published guidelines or recommendations, such as the high prevalence of low‐yielding radiographic imaging and opiate prescription.

Aged care residents had more comorbidities and higher rates of polypharmacy than community‐dwelling older adults, in line with current non‐LBP literature [17, 21]. Similarly, aged care residents also presented with more baseline analgesic medications than community‐dwelling older adults, as previously reported in the non‐LBP literature [22]. A higher prevalence of recent reported falls was also observed in the aged care group. The rates of a serious LBP diagnoses, such as a fracture following fall, observed in both aged care residents and an age and sex‐matched sample of community‐dwelling older adults were low, and lower in our study than reported previously (3% vs*.* 4% to 5%) [5, 14]. This is a reassuring finding and gives weight to the potential for future research to support hospital avoidance or management minimisation strategies, such as minimising low‐value care.

Consistent with recent research on all adult LBP presentations to the ED, both groups in this study showed concerningly high prevalence of hospital admissions, diagnostic testing and use of opioid medications [14, 23]. Themed analysis of clinicians' reasons for admission revealed admissions were mainly for further diagnostic investigations and ongoing analgesia across both groups. Interestingly, community‐dwelling older adults were more likely to be admitted to hospital for Allied Health input with more people being referred for Physiotherapy consults whilst in the ED than aged care residents. This result may reflect the higher levels of support available to those in residential aged care following discharge when compared to community dwelling older adults. Services, such as the Acute Geriatric Outreach Services [24] and Hospital in the Nursing Home programmes, have been shown to reduce hospital admissions from the ED, and are two such examples of support services available to patients in aged care facilities only. By facilitating medication review and optimisation as well as the organisation of pathology and radiology services in the community, these services have positive impacts in reducing hospital admissions of aged care residents [25]. Likewise, both Allied Health‐led [26] and Nurse‐led interventions [27] prioritising early functional assessment and organisation of relevant community follow‐up services were shown to significantly reduce avoidable hospital admissions from the ED for those older adults living in the community.

Prevalence of opioid administration in the ED for LBP in both aged care residents (81%) and community‐dwelling older adults (91%) within this study were considerably higher than previous literature reporting on opiate administration for LBP in Australia (70%) [14], United States (62%) [28] and Canada (35%) [29]. However, rates were similar to results reported for older adults presenting to the ED with pain for any reason (80%) [30]. Whilst conjecture still exists as to the role of opiate medication in the ED management of LBP across all age groups [31], careful consideration should be made for the older adult with LBP. Age‐relevant risks for the older adult, such as sedation as well as increased risk of falls and fractures [32, 33], suggest this area of LBP management may warrant further research and awareness.

Lumbar spine medical imaging for LBP has received a lot of attention in ‘low‐value care’ and ‘choosing wisely’ literature [34]. In our study, a large proportion of patients received at least one form of lumbar spine imaging with a preference for aged care residents in comparison with community dwelling older adults (61% vs. 50%). These values were higher than previously reported (49%) in people aged 70 years or older presenting to ED with LBP [35]. About one in three patients in both groups received advanced imaging (CT or MRI). Most imaging findings were classified as longstanding findings with few reports of acute findings for both aged care residents (15%) and community‐dwelling older adults (13%). Previous research has shown the prevalence of MRI and CT imaging within the ED as being higher in the older population (26%) than in adults under the age of 65 (11%), reasons for which are proposed as being due to a higher risk of bony injury from low‐trauma mechanisms in older people [5]. Our study found only 3% of all presentations in this age population had a newly diagnosed spinal fracture at time of ED assessment, although this number is higher than found in previous studies including all age groups [5]. This study found many older adults had chronic findings in their imaging such as previously identified vertebral fractures, disc degeneration and osteophytic bony abnormalities, as would be expected in this population. There are currently no age‐standardised clinical practice guidelines for ED management of LBP, nor recommendations for optimal usage of imaging for the older adult with LBP in ED.

Community‐dwelling older adults who lack social supports typically have greater utilisation of ED services and are at increased risk of hospital admission [36, 37, 38]. This study revealed 20% of community‐dwelling older adults included in this study live alone and 34% of the group engaged formal support services. Of these community‐dwelling older adults, those with formal support services were less likely to be admitted to hospital (26%) as a result of their ED visit for LBP compared to those without formal supports (36%). This may reflect a reluctance of clinicians to discharge patients with LBP until pain and functional outcomes demonstrate improvement or—perhaps more likely—a reluctance to discharge patients who have insufficient levels of social support. Further research into the probability of hospital admission for LBP and levels of social support is warranted.

The main limitation of this study was the relatively small sample of aged care residents attending ED with the primary complaint of LBP within the recruitment period (*N* = 64). A larger sample across multiple jurisdictions would improve generalisability and the power of hypothesis tests. Information on outcomes extending past this current presentation would be beneficial, such as representation rates and subsequent healthcare utilisation costs.

## 5 Conclusion

To our knowledge, this is the first study comparing presenting characteristics and management of aged care residents and community‐dwelling older adults presenting to ED for LBP. This study found these two groups differed on baseline comorbidity, polypharmacy and baseline analgesic medication presentation characteristics. Both groups had high rates of hospital admissions, opioid use and lumbar spine imaging when compared to an adult population across all ages. Interestingly, a greater number of aged care residents were admitted for ongoing analgesia or further diagnostic tests while a greater number of community‐dwelling older adults were admitted for Allied Health input. Despite the very high rates of lumbar spine imaging, there was low yield of acute findings and almost all people from both groups were diagnosed with non‐specific LBP rather than LBP with radicular symptoms or serious/sinister pathology. Whilst this study does not establish causation for the higher prevalence of admissions, opiate use and imaging in the older population presenting to the ED with LBP, it does highlight that differences exist when managing the older adult in this population. This study should guide future investigations into the consideration of age‐appropriate clinical practice guidelines for older adults presenting to ED with LBP. This may have important implications for health service delivery and development of alternative clinical care pathways tailored to the needs of aged care residents and community‐dwelling older adults.

## Conflicts of Interest

The authors declare no conflicts of interest.

## Supporting information

Supporting information

## Data Availability

The data that support the findings of this study are available on request from the corresponding author. The data are not publicly available due to privacy or ethical restrictions.
