# Supplementary material for: Low Back Pain Presentation and Management at the Emergency Department: Differences Between Older Adults Residing in the Community and Aged Care Homes
Source: J Eval Clin Pract. 2025 Apr 11;31(3):e70088. doi: 10.1111/jep.70088 (PMC11987480; doi:10.1111/jep.70088)
Supplement: Supplementary file 1 — Supporting information [file JEP-31-0-s001.docx]

**Appendix 1**

Supplementary file 1. SNOMED CT-AU (EDRS) codes related to low back pain presentations

Description Codes

**Low back pain with non-specific cause**

Acute low back pain (finding) 278862001

Back pain complicating pregnancy (disorder) 91957002

Backache (finding) 161891005

Blunt injury to back (disorder) 424270008

Chronic back pain (finding) 134407002

Chronic low back pain (finding) 278860009

Coccyx sprain (disorder) 209571002

Complaining of low back pain (finding) 161894002

Degeneration of lumbar intervertebral disc (disorder) 26538006

Displacement of lumbar intervertebral disc without myelopathy (disorder) 20021007

Exacerbation of backache (finding) 135860001

Low back pain (finding) 279039007

Low back strain (disorder) 300956001

Lower back injury (disorder) 282766005

Lumbar spondylosis (disorder) 239880009

Lumbar sprain (disorder) 209565008

Mechanical low back pain (finding) 279040009

Pain in the coccyx (finding) 34789001

Sacral back pain (finding) 61486003

Spasm of back muscles (finding) 203095000

Sprain of ligament of lumbosacral joint (disorder) 209548004

Stiff back (finding) 249921008

Strain of back muscle (disorder) 262965006

Strain of tendon of back (disorder) 262975009

**Low back pain with neurological signs and symptoms**

Acute back pain with sciatica (finding) 247366003

Acute sciatica (disorder) 307176005

Chronic sciatica (disorder) 307177001

Injury of lumbar nerve roots (disorder) 24300005

Injury of sciatic nerve (disorder) 86269002

Lumbago with sciatica (finding) 202794004

Lumbago-sciatica due to displacement of lumbar intervertebral disc (disorder) 46960006

Lumbar disc prolapse with radiculopathy (disorder) 202735001

Lumbar radiculopathy (disorder) 128196005

Sciatica (disorder) 23056005

Spinal stenosis of lumbar region (disorder) 18347007

**Low back pain due to serious pathology**

Abscess of back (disorder) 309083007

Abscess of back, except buttock (disorder) 19284003

Cauda equina syndrome (disorder) 192970008

Closed fracture lumbar vertebra (disorder) 207957008

Collapse of lumbar vertebra (disorder) 308758008

Compression fracture of lumbar spine (disorder) 426646004

Concussion and edema of lumbar spinal cord (disorder) 212360005

Contusion of back (disorder) 11437003

Contusion of lower back (disorder) 284062002

Crush fracture of lumbar vertebra (disorder) 281933002

Disc prolapse with myelopathy (disorder) 202728009

Discitis (disorder) 2304001

Fracture of coccyx (disorder) 125871005

Fracture of lumbar spine (disorder) 125608002

Fracture of lumbar spine and/or pelvis (disorder) 207986006

Injury of cauda equina (disorder) 230614002

Lumbar disc prolapse with myelopathy (disorder) 202731005

Multiple fractures of lumbar spine and/or pelvis (disorder) 207993005

Open dislocation of coccyx (disorder) 44237008

Open fracture of lumbar vertebra with spinal cord injury (disorder) 48956000

Open fracture of sacrum AND/OR coccyx with spinal cord injury (disorder) 65491009

Traumatic dislocation of joint of lumbar vertebra (disorder) 129166009

SNOMED CT-AU (EDRS), Systematized Nomenclature of Medicine – Clinical Terms – Australian

Version (Emergency Department Reference Set). Supplementary material *BMJ Qual Saf* Ferreira
